# Research on Performance Improvement of Emulsified Asphalt Mixture Based on Innovative Forming Process

**DOI:** 10.3390/ma17061430

**Published:** 2024-03-21

**Authors:** Ke Xiao, Xin Qu, Yong Jiang, Wenyang Yun, Pengfei Zheng, Weicheng Li

**Affiliations:** 1 School of Highway, Chang’an University, Xi’an 710064, China; xiaoke@chd.edu.cn (K.X.); 2022121184@chd.edu.cn (W.Y.); 2023221323@chd.edu.cn (P.Z.); 2 Ankang Transportation Investment Construction Co., Ltd., Ankang 725000, China; aa564571546@163.com (Y.J.); theprc@163.com (W.L.)

**Keywords:** pavement engineering, new mixing technology, adhesion, CT scanning, porosity, surface tension

## Abstract

Bulk density and porosity have great influence on the technical performance of an emulsified asphalt mixture, so in order to enhance the strength of the asphalt mixture, bulk density should be improved and porosity should be reduced. Considering the forming process of the emulsified asphalt mixture, the decrease in porosity can ensure the state of the mixture. In order to reduce the porosity of the emulsified asphalt mixture, an innovative forming process is proposed to improve the performance of the emulsified asphalt mixture, and its strength formation mechanism is explored in this paper. Three groups of emulsified asphalt mixtures (ARC-8 + SBR, SMA-5 + EVA, SMA-5 + SBR) were prepared by a conventional mixing process and novel mixing process. Marshall test of the emulsified asphalt mixture, CT scanning test of the emulsified asphalt mixture, workability test and analysis were manufactured and tested. The results show that, compared with conventional methods, the innovative forming method can increase the bulk density of the mixture and reduce the porosity, and thus improve its technical performance. The reason is that most of the water in the mixture of the innovative forming method sticks to the outer surface of the fine aggregate, and the water is more easily discharged. Secondly, the fine aggregate of the innovative forming method is directly mixed with the emulsion, and the volume is smaller. The emulsion wraps the fine aggregate in it due to the surface tension, which enhances the adhesion effect, thus improving the strength of the mixture.

## 1. Introduction

Since the 19th National Congress of China, achieving carbon peaking and carbon neutralization is an inherent requirement for China’s high-quality economic development, as well as a complex and arduous long-term project. The transportation industry is one of the important areas of carbon emissions. In view of the requirements of various policies on “carbon peak and neutrality targets” transportation, the state has actively promoted the development of scientific research on green, low-carbon and resource-saving road materials and construction techniques. It has also promoted the construction of green highways and carried out a number of typical demonstration projects. Moreover, it has vigorously promoted the application of new eco-friendly structures, materials, processes and equipment [1,2]. Currently, 99.4% of our road network has entered the maintenance phase. Emulsified asphalt offers several advantages over other maintenance materials, primarily due to its versatile applications and suitability for various road maintenance projects. In comparison to diluted asphalt, it promotes energy efficiency and emission reduction while also providing convenient usage for localized repairs and maintenance tasks. Notably, it demonstrates excellent effectiveness in rehabilitating highway pavements. Years of research and practice have shown that emulsified asphalt can play a very significant role in highway maintenance, and its cost is very low, so it is very suitable for nationwide promotion and application [3].

However, water damage is often caused by insufficient adhesion between emulsified asphalt and aggregate in actual engineering, which leads to spalling of the pavement layer and various pavement diseases. In view of the shortcomings of general emulsified asphalt, such as low bonding degree, poor flexibility and insufficient strength [4,5,6], at present, domestic scholars [7,8] have proved that water-based epoxy resin as an anti-spall agent can improve the adhesion between emulsified asphalt and aggregate through theoretical and boiling tests. However, in order to solve the defects of the boiling method, domestic and foreign scholars [9,10,11,12,13] introduced the surface energy theory and studied the adhesion of ordinary asphalt and SBR modified emulsified asphalt mixed with water-based epoxy resin with aggregate based on parameters such as adhesion work [14,15]. The strength, high temperature stability and water stability of the emulsified asphalt mixture were improved significantly by the addition of water-based epoxy resin. The low temperature performance of the mixture is comparable to that of the hot mix asphalt mixture, which has advantages. Due to the influence of more voids formed by the volatilization of water in the mixture, the fatigue performance of the mixture is significantly reduced compared with that of the hot-mixed asphalt mixture [16]. Similarly, cement content has the greatest influence on the demulsification time of emulsified asphalt and the initial and final strength of cold replenishment of emulsified asphalt, and the ratio of oil to stone has the greatest influence on the penetration force of cold replenishment of sulfur-aluminate cement-emulsified asphalt [17].

Due to the different types of emulsified asphalt, mixture gradation, cement amount and mixing water consumption, the strength of the mixture will show three trends. (1) Due to the mismatching of emulsifier formula, the demulsification speed of emulsified asphalt is too fast, the mix workability is poor, and the working time is insufficient, resulting in demulsification before spread molding on-site. (2) Due to the mismatching of emulsifier formula, the demulsification speed of emulsified asphalt is too slow, resulting in a long curing time, and the strength-building process is too slow, which will affect the superstructure of the paving and construction progress. (3) Regarding the ideal demulsification speed of emulsified asphalt, before the mixture is spread, the emulsified asphalt is not yet demulsified, and the mixture has good workability. In the process of spreading and rolling, the emulsified asphalt begins to demulsify, the water is extruded, the growth rate of the strength of the mixture begins to accelerate, the curing time is extended, and the strength of the mixture is stabilized at a certain level [18].

In order to enhance the adhesion performance of emulsified asphalt and strengthen the emulsified asphalt mixture, this study fabricated various epoxy-based emulsified asphalts and optimized the conventional mixing process for emulsified asphalt. The study was conducted under experimental conditions where the emulsion dosage and mixing water volume were set at their optimal proportions, as per the conventional molding method. By comparing the performance of emulsified asphalt mixture specimens formed using both the conventional and novel mixing processes, the internal mechanism of the novel mixing process in enhancing emulsified asphalt mixture performance was analyzed and validated. This was done by examining the interface affinity between asphalt and aggregate from both macroscopic and microscopic perspectives, such as through the porosity analysis of the emulsified asphalt mixture via CT scan and the evaluation of workability in emulsified asphalt.

## 2. Materials and Methodology

### 2.1. Test Raw Materials

In this study, the raw materials for various emulsified asphalt formulations primarily consist of: SK-90 matrix asphalt, CCR emulsifier, SBR latex, and Ethyene-vinyl acetate copolymer (EVA) emulsion. The characteristics of each constituent are as follows.

#### 2.1.1. Base Asphalt

The Basic properties of matrix asphalt are shown in the Table 1.

#### 2.1.2. Emulsifier

The properties of emulsifier in this work are shown in Table 2.

#### 2.1.3. Modifier

##### SBR

**Figure 1:**
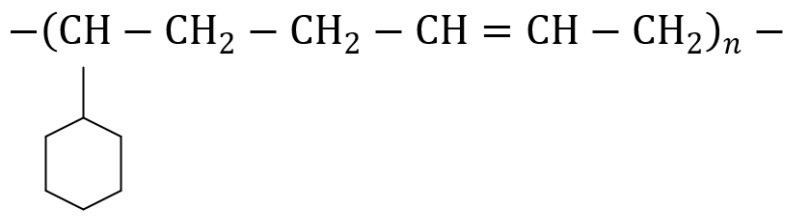
The molecular formula of SBR.

SBR is a linear copolymer of butadiene and styrene. Its molecular formula can be abbreviated as shown in Figure 1.

Due to the high relative molecular weight of SBR, the interaction force between molecules is also large, and the molecules are intertwined, so SBR has good viscoelasticity. It can be concluded from the previous test results that SBR can obviously improve the high and low temperature stability of emulsified asphalt evaporation residue, especially the low temperature performance [19].

##### EVA

**Figure 2:**
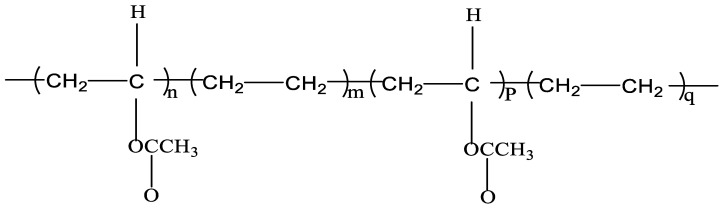
The molecular formula of EVA.

EVA is short for vinyl acetate–vinyl copolymerization emulsion. It is a kind of polymer emulsion which is formed by the copolymerization of vinyl acetate and vinyl monomer with other auxiliary materials through emulsion polymerization. Its molecular formula is shown in Figure 2 [20].

EVA emulsion also has permanent flexibility. The EVA emulsion can be regarded as an internal plasticizing product of polyvinyl acetate emulsion. As it introduces ethylene molecular chain into polyvinyl acetate molecules, the acetyl group produces discontinuity and increases the rotational freedom of a polymer chain. The spatial obstruction is small, the main polymer chain becomes soft, and plasticizer migration does not occur, ensuring the permanent softness of the product.

### 2.2. Mixing Process Optimization Test

The modified Marshall method of a cationic emulsified asphalt mixture was used to design the mix ratio.

(1) Determine the optimal amount of emulsified asphalt

P = 0.06a + 0.12b + 0.20c

where: P is the percentage of emulsion in dry mass of aggregate; 

a is the percentage of aggregate greater than 2.5 mm in the total mass of all aggregate;

b is the percentage of aggregate in 2.5–0.074 mm in the total mass of all aggregate;

c is the percentage of aggregate less than 0.074 mm in the total mass of all aggregate.

(2) Determine the best water consumption

The absolute water consumption of the dense graded mixture should be 5.5% to 7.0% of the total mass of the mineral, and then subtract the water in the emulsified asphalt emulsion and the water in the aggregate; that is, the mixing water.

#### 2.2.1. Conventional Mixing Process of Emulsified Asphalt Mixture

**Figure 3:**
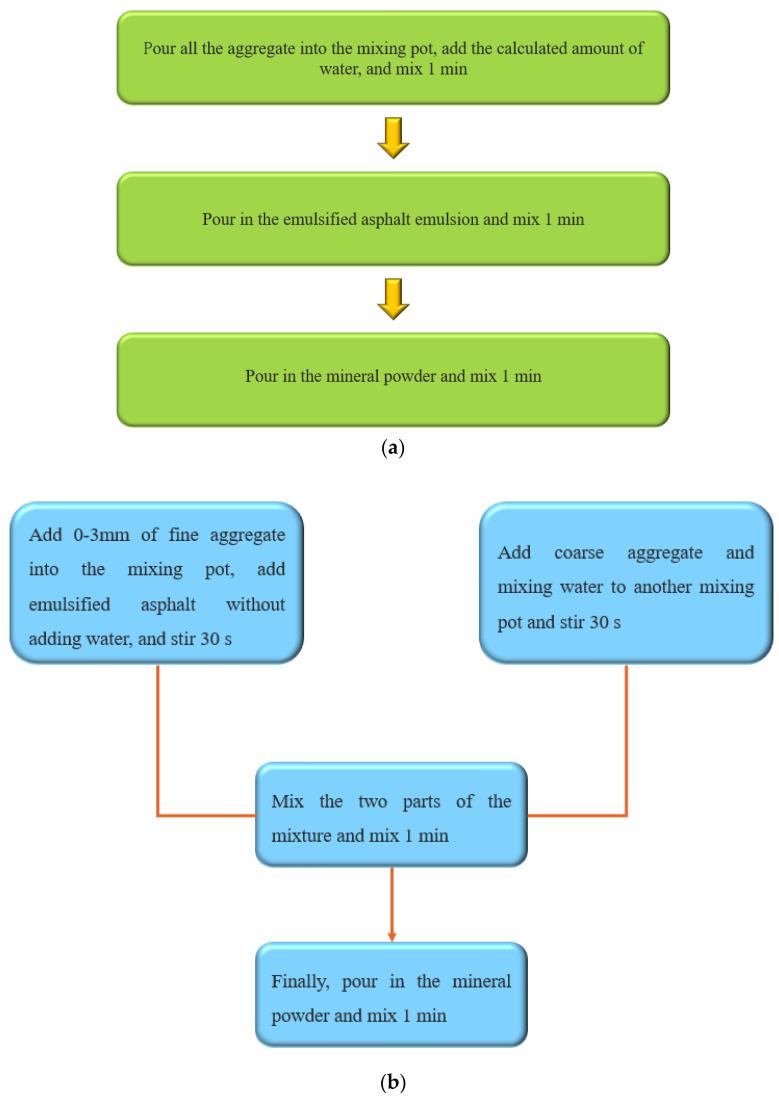
Mixing process diagram: (**a**) conventional way, (**b**) novel way.

The conventional mixing method of the emulsified asphalt mixture is shown in Figure 3a.

Striking times and curing conditions were carried out according to the modified Marshall molding method.

#### 2.2.2. Novel Mixing Process of Emulsified Asphalt Mixture

The dosage of the emulsion and mixing water was the optimal proportion designed in the conventional molding method. The new mixing method is shown in Figure 3b.

Striking times and curing conditions were consistent with the conventional methods.

The test is divided into three groups. The specific materials are shown in Table 3.

Among them, the base asphalt of emulsified asphalt A is SK-90, and the emulsifier type is CCR, which is processed by colloid mill. Emulsified asphalt B is the finished emulsified asphalt produced in Yunnan.

## 3. Results and Discussion

### 3.1. Performance Comparison between Different Mixing Processes

**Figure 4:**
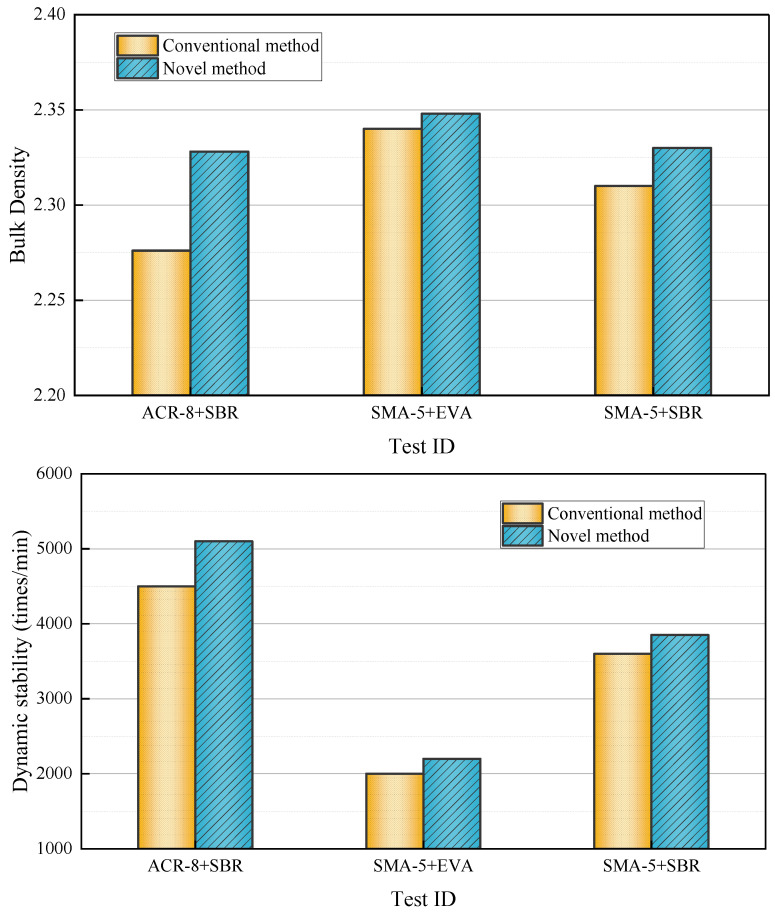

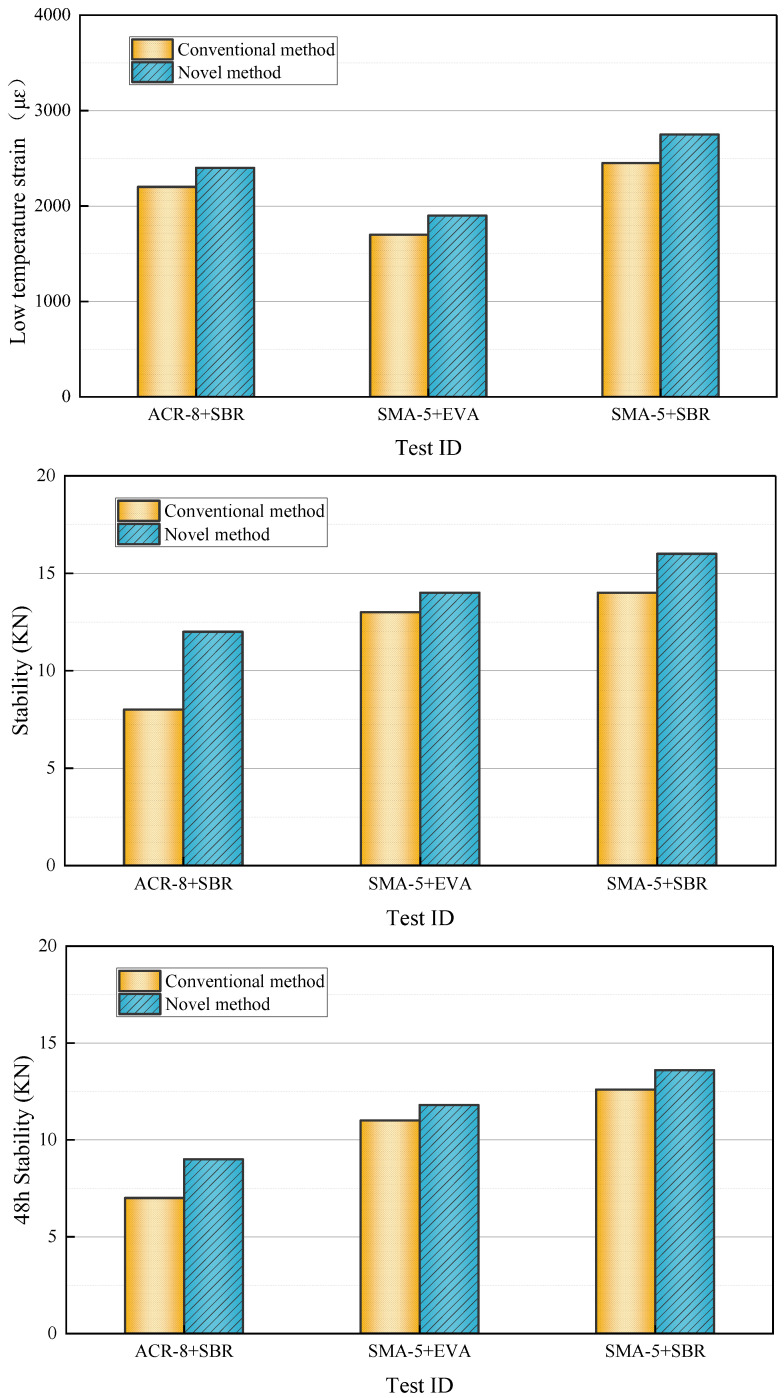
The test results of different groups.

The emulsified asphalt mixtures mentioned above were prepared using both conventional and novel mixing processes, respectively. Each group contains 4 specimens. A comparative analysis of their technical properties was conducted, and the corresponding test results are presented in Figure 4.

**Figure 5:**
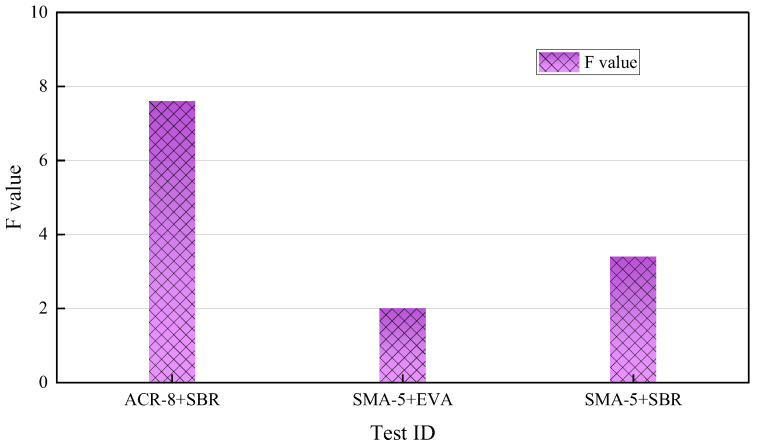
Density variance of the emulsified asphalt mixture.

Because the conventional method and the new method use the same mix ratio, the most fundamental reason for the performance difference is the change in the porosity of the mixture. The decrease in the porosity of the mixture formed by the new method leads to an increase in its density and the improvement of its road performance. The variance analysis of emulsified asphalt mixture density is shown in Figure 5.

**Figure 6:**
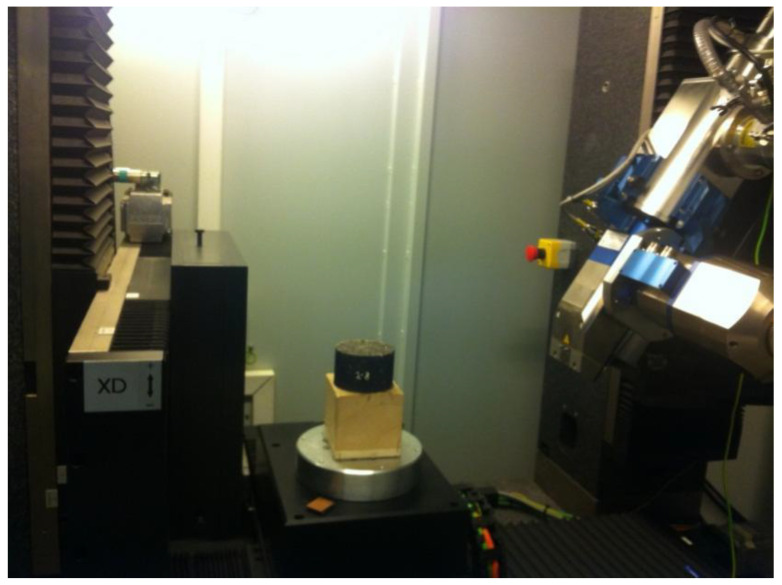
CT scanning test.

Based on the variance analysis results, if the F value is close to 1, the difference between the mean values of each group is not statistically significant. On the other hand, if the F value is much larger than 1, the difference between the mean values of each group is statistically significant. By comparing the data results in Figure 6, it can be seen that the F values of the three groups are much higher than 1, indicating that there is a significant difference in the density of the mixture formed by the conventional method and the new method, and the density of the mixture formed by the new process is significantly increased.

The increase in the density of the first group is mainly due to the large porosity of the ARC-8 graded mixture and the fact that the new forming method has a large space to reduce the porosity, while the porosity of the other two groups of the SMA-5 graded mixture is already small, so the increase in amplitude is slightly smaller. Comparing the density data of the last two groups reveals that the third group has higher evaporation residue content and higher viscosity of the emulsified asphalt emulsion, resulting in higher porosity after compaction, so there is more room for density improvement than in the second group.

As a whole, it can be concluded that the porosity of the emulsified asphalt mixture decreases by the innovative forming method, the density increases, and the high temperature performance, low temperature performance and water damage stability performance are improved to a certain extent.

### 3.2. Adhesion Analysis of Asphalt and Fine Aggregate

The angle of aggregate is obvious, the crushing value is small and the adhesive property is good. However, there is more flat aggregate material, the fine material content is large and its adhesion is poor. The essence of adhesiveness between aggregate and asphalt is the interface affinity of the two materials, which refers to surface tension, molecular attraction (van der Waals force), mechanical adhesion and chemical reaction attraction.

When mixing the cationic emulsified asphalt mixture, it is generally required to add water first and then emulsified asphalt for mixing. The aim is to improve the wetting performance of asphalt on the ore surface, which is conducive to the wetting and intrusion of asphalt into the cracks and pores on the ore surface, which can not only shorten the distance between the two phases, but also significantly increase the contact area and form a higher strength. In the innovative forming method, the fine aggregate is mixed directly with the emulsion. Due to the large specific surface area of the fine aggregate, the emulsified asphalt wrapped in it will quickly demulsify enough water to moisten the surface of the ore. Therefore, the innovative forming method can not only achieve the same wetting conditions to ensure adhesion, but also shorten the distance between the emulsion and the aggregate.

In the innovative forming process, due to the miniaturization of the size of the fine aggregate, the influence of gravity is very small, and the surface tension plays a great role between the contact surfaces. Liquid surface tension is caused by the imbalance of molecular cohesion at the liquid surface, resulting in the liquid surface shrinking and having the properties of an unfolded elastic film. The emulsion will shrink into a ball due to the surface tension, and the fine aggregate will just be wrapped in it.

### 3.3. CT Scanning Porosity Analysis of Emulsified Asphalt Mixture

After CT scanning the specimen, IPP 6.0 was used to calculate the porosity of the cold regenerated mixture. See Figure 6 for the CT scanner.

**Figure 7:**
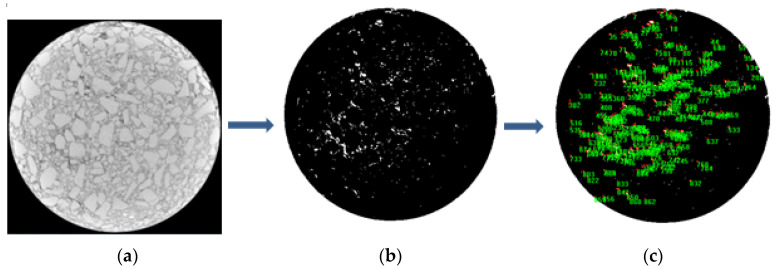
CT scanning analysis diagram. (**a**) CT scanning section, (**b**) pore part screened out, (**c**) pore area calculation.

Then, Image-Pro Plus 6.0 software was used to analyze the CT scanning images as shown in the Figure 7.

Through IPP software, after setting the maximum pore size, the porosity ratio is calculated as shown in Table 4.

Through the test method of CT scanning, it was further confirmed that the void ratio of the Marshall mixture formed by the new mixing method was smaller.

In addition, the porosity calculated by CT scanning was smaller than that obtained by the dry surface method because only the closed porosity could be calculated by the IPP software. However, in the surface dry method test, because the towel could not wipe the water in some deep open pores, the porosity calculated by the surface dry method included the closed porosity + partial open porosity. Therefore, the calculation result of table dry method is larger than that of IPP image processing.

### 3.4. Workability Analysis of Emulsified Asphalt Mixture

**Figure 8:**
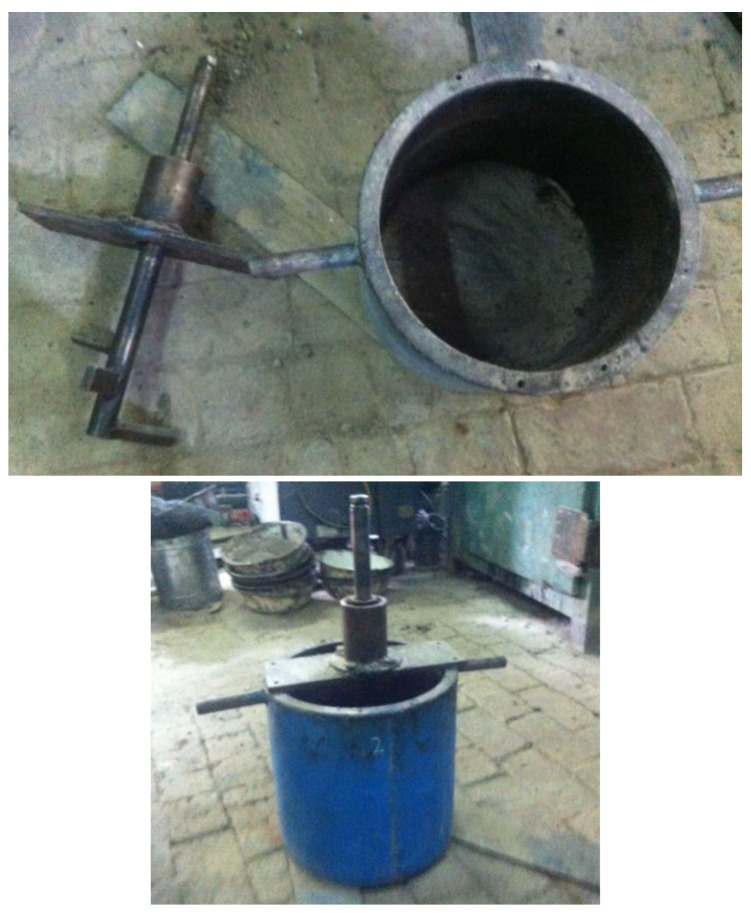
Workability equipment.

Workability equipment and torque wrench were used to test the mixture. The workability meter is shown in Figure 8.

The workability index in this paper is the torsion force on the blade in the stirring process, so the greater the workability value, the greater the force on the blade, indicating that the more difficult the asphalt mixture is to mix, the worse the workability. The stirring rate of the torque wrench in this test is 3 s/RPM, and the workability results are shown in Table 5.

Although there is basically no difference in the appearance of the mixture mixed by the conventional method and the new method, it can be seen from the workability test results in Table 3 that the torque value of the mixture in the novel mixing process is smaller than that of the conventional one because the two mixing processes add the same amount of water. In the novel mixing process, because the fine aggregate is coated with the asphalt emulsion, the added water is not absorbed. So, there is more free water in the novel mixing process, which indicates that the new mixing method has better workability.

### 3.5. Analysis of Strength Formation Mechanism of Novel Mixing Process

(a) Mixing process

Water lubricates the aggregate during mixing. In the conventional process, water is added first and then stirred. In this way, due to the lubrication effect of water, emulsified asphalt can be more fully and evenly distributed in the mixture during mixing so as to ensure the uniformity and stability of the asphalt film. Although the new forming process involves stirring without adding water in the first step, it ensures the uniform distribution of the emulsified asphalt because only the fine aggregate with a particle size of 0–3 mm is added, and the emulsified asphalt begins to mix in relatively large quantities.

(b) Compaction and curing process

**Figure 9:**
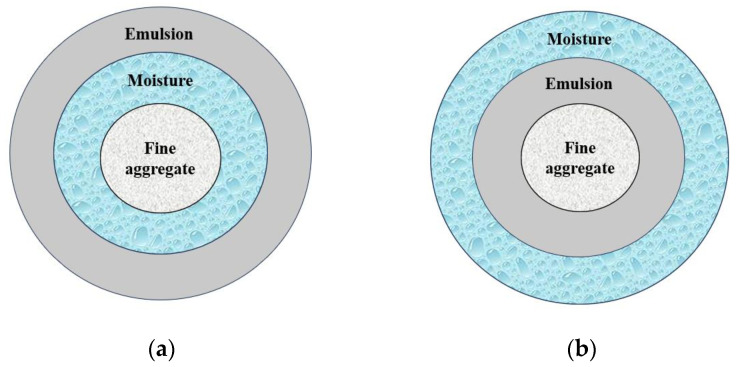
Moisture position in different mixing methods. (**a**) Conventional mixing method, (**b**) novel mixing method.

In the compaction process, the emulsified asphalt emulsion and water act as lubricant, which is conducive to the compaction of the mixture. In the curing stage, the water dispersed in the emulsified mixture cannot be immediately discharged, and most of the water is free and occupies the space of the mixture. The drainage behavior in the curing process increases the porosity of the mixture, resulting in a reduction in mixture density and strength. In the mixture specimen made by the conventional molding process, all the water is wrapped between the emulsified asphalt and aggregate, which is difficult to volatilize, resulting in a void ratio that is too large after curing. In the mixture formed by the new process, because the water of the fine aggregate part is attached to the outermost part, it is easier to discharge the mixture; that is, it can reduce the porosity and further increase the density. The simulation diagram is shown in Figure 9.

(c) The formation process of the strength of the mixture

With the discharge of water, the viscous force and internal friction of the mixture are increased. So, water also affects the speed at which the mixture gains strength. The new forming process allows the water to be discharged more easily, so its strength is formed more quickly.

## 4. Construction Site Process Suggestions

In this section, the application of the innovative forming method in construction projects will be considered and discussed. In engineering practice, emulsified asphalt mixtures with better technical properties can be produced by innovative forming methods. However, due to the large investment in equipment during site construction and the high cost of adding a mixing pot, it is necessary to propose the corresponding mixing process for the innovative forming equipment at the current stage of the construction site. Firstly, only add the fine aggregate with a particle size of 0~3 mm into the mixing pot, then directly add the emulsified asphalt without adding water, and stir for 30 s.

Secondly, add coarse aggregate to the mixing pot, and then use the nozzle to spray the corresponding mixing water into the coarse aggregate, and stir for 1 min.

Finally, add mineral powder and stir for 1 min again.

## 5. Conclusions

The emulsified asphalt mixture with the innovative forming method can improve the bulk density of the mixture and reduce the porosity of the emulsified asphalt mixture so as to improve its technical performance.

The fine aggregate of the innovative forming method is directly mixed with the emulsion. Because the specific surface area of the fine aggregate is large, the moisture generated by the emulsion adhering to the surface is enough to wet the surface and produce good adhesion.

In the mixture formed by the novel method, most of the water adheres to the outer surface of the fine aggregate, the water is easier to discharge, the density of the mixture increases, and the porosity decreases.

In the curing stage of the mixture formed by the novel method, the water discharge speed is fast and strength is quickly gained.

Regarding the fine aggregate and emulsified asphalt in the mixing process, the innovative forming method of the fine aggregate does not mix with water, the volume is smaller, and the emulsion resulting from the surface tension of the fine aggregate wrapped in it enhances the “adhesion” effect.

## Figures and Tables

**Table 1:** Basic properties of matrix asphalt.

Model	Penetration of Needle (25 °C/0.01 mm)	Softening Point (°C)	Elongation (15 °C/cm)
SK-90	88.7	46.0	>150

**Table 2:** Properties of emulsifiers.

Specifications and Models	Effective Content	Appearance	Charge	PH Value	Stability of Mixing	Degree of Solubility
CCR	60 ± 2%	Brown liquid	+	5~7	Slow crack	99.8%

**Table 3:** The specific materials of each test.

Test ID	Gradation	Modifier
A	ARC-8	SBR
B	SMA-5	EVA
C	SMA-5	SBR

**Table 4:** Porosity calculated by IPP software.

Test Grouping	ARC-8 + SBR Emulsion A		SMA-5 + EVA Emulsion B		SMA-5 + SBR Emulsion B	
Forming technology	Conventional technology	Novel technology	Conventional technology	Novel technology	Conventional technology	Novel technology
Porosity (%)	7.202	5.970	4.345	4.114	4.650	3.900

**Table 5:** Torque value of workability test.

Test Grouping	ARC-8 + SBR Emulsion A		SMA-5 + EVA Emulsion B		SMA-5 + SBR Emulsion B	
Forming technology	Conventional technology	Novel technology	Conventional technology	Novel technology	Conventional technology	Novel technology
torque(N.m)	19.95	18.36	13.45	12.30	14.42	13.25

## Data Availability

Data are contained within the article.
